# Attitudes, Motivation, and Predictors of Influenza Vaccination Uptake Among Primary Healthcare Professionals in Greece

**DOI:** 10.3390/vaccines14060500

**Published:** 2026-06-02

**Authors:** Isidoros Kougioumtzoglou, Evangelia-Georgia Kostaki, George Soulis, Nikos Selekos, Areti-Dimitra Koulouvari, Dimitrios Kouvelas, Nikos Maniadakis, Areti Lagiou

**Affiliations:** 1 Laboratory of Hygiene and Epidemiology, Department of Public and Community Health, School of Public Health, University of West Attica, 12241 Athens, Greece; hv17083@uniwa.gr (N.S.); adkoulouvari@uniwa.gr (A.-D.K.); alagiou@uniwa.gr (A.L.); 2 Department of Hygiene, Epidemiology and Medical Statistics, Medical School, National and Kapodistrian University of Athens, 15784 Athens, Greece; ekostakh@med.uoa.gr; 3 Hellenic Society for the Study and Research of Ageing, Scientific Society, 11528 Athens, Greece; geosoulis@yahoo.com; 4 Laboratory of Clinical Pharmacology, Faculty of Medicine, School of Health Sciences, Aristotle University of Thessaloniki, 54124 Thessaloniki, Greece; kouvelas@auth.gr; 5 Department of Public Health Policy, School of Public Health, University of West Attica, 12241 Athens, Greece; nmaniadakis@uniwa.gr

**Keywords:** influenza vaccination, vaccine uptake, primary healthcare, health professionals, vaccination behavior, predictors, cross-sectional study, preventive health, Greece

## Abstract

*Background*: Influenza vaccination uptake among healthcare professionals remains suboptimal despite their key role in influencing public vaccination behavior. This study investigated motivational and behavioral determinants of influenza vaccination uptake and advocacy among primary healthcare professionals in Greece. *Methods*: A cross-sectional study was conducted among 304 physicians and pharmacists using an anonymous online questionnaire. Vaccination uptake (2023–2024 season and annual) and motivational and advocacy constructs were assessed using the validated MoVac-flu and MovAd scales. Factor structure was evaluated using confirmatory and exploratory factor analyses. Multivariable logistic regression models were applied to identify predictors of vaccination uptake. *Results*: The study sample consisted of 304 healthcare professionals of whom 61.2% were physicians and 38.8% were pharmacists. More than half of the participants were female (52.6%) and aged 41–60 years (57.6%). Influenza vaccination uptake was 77.6% for the 2023–2024 season and 75.3% for annual vaccination. A two-factor structure was identified for the MoVac-flu scale (F1: Vax Self-Care, F2: Vax Awareness), whereas a four-factor structure was identified for the MovAd scale (F1: Vax Communication, F2: Vax Influence, F3: Vax Confidence, F4: Vax Choice). The overall scales demonstrated high internal consistency, while most subscales showed satisfactory to high reliability. Motivation toward influenza vaccination and vaccination advocacy were high among the participants. Vaccinated participants demonstrated higher motivation and vaccination advocacy scores compared with non-vaccinated participants. In multivariable analyses, higher scores on Vax Self-Care (aOR = 3.22, 95% CI: 2.08–4.96, *p* < 0.001) and Vax Communication (aOR = 1.64, 95% CI: 1.14–2.34, *p* = 0.007) subscales, reflecting higher motivation and vaccination advocacy, respectively, as well as male sex (aOR = 2.35, 95% CI: 1.14–4.83, *p* = 0.020) were associated with higher odds of annual vaccination. Higher scores on the Vax Self-Care subscale (aOR = 3.66, 95% CI: 2.33–5.77, *p* < 0.001) were also found to be associated with higher odds of 2023–2024 vaccination uptake, as well as living with vulnerable individuals (aOR = 2.95, 95% CI: 1.18–7.38, *p* = 0.020). *Conclusions*: Influenza vaccination uptake among primary healthcare professionals in Greece was relatively high; however, it was strongly driven by intrinsic motivational factors, particularly the perceived personal and public health benefits of vaccination. Communication-related competencies also independently contributed to vaccination behavior, highlighting the link between professional practice and personal uptake. In contrast, household-related contextual characteristics, such as cohabitation with vulnerable individuals, appeared to exert a less consistent influence on vaccination behavior. These findings suggest that interventions focusing on strengthening intrinsic motivation and communication skills may contribute to sustained improvements in both vaccination uptake and advocacy among healthcare professionals.

## 1. Introduction

Vaccination is one of the most effective strategies for the prevention of communicable diseases, with the World Health Organization estimating that 3.5–5 million deaths could be prevented annually through immunization [1]. Influenza remains one of the most common infectious diseases worldwide, and seasonal influenza vaccination is considered the most effective evidence-based intervention for reducing influenza-related morbidity and mortality [2]. In addition to its clinical benefits, vaccination represents a cost-effective primary prevention strategy applicable across both high- and low-to-middle-income settings [3].

Despite these benefits, influenza continues to pose a significant public health burden in Europe, where vaccination coverage and acceptance remain suboptimal. In Greece, although vaccination uptake has shown some improvement in recent years, it remains below recommended levels [4]. Influenza activity peaks during the winter months and disproportionately affects vulnerable populations, particularly older adults [5]. Mortality estimates suggest that approximately 25 deaths per 100,000 population are attributable to influenza annually, with the majority occurring in individuals over 65 years of age [6]. Vaccination uptake in Greece remains suboptimal across key population groups, particularly among healthcare workers (36–57%) and high-risk populations (~50–55%) [7], despite evidence from surveillance data indicating low vaccination coverage among severe or confirmed cases in earlier years (<10% in 2015–2016) [8].

Vaccine hesitancy has been identified as a key contributor to low vaccination uptake. This phenomenon is well documented in both the Greek and broader European context, affecting diverse population groups. For example, studies in Greece have reported high levels of hesitancy toward COVID-19 vaccination, driven by concerns about safety, low perceived risk, prior infection, and misinformation. Similar patterns of hesitancy have been observed historically in the context of influenza vaccination, including during the 2009 pandemic, where low vaccination uptake contributed to increased disease burden and mortality [9,10,11].

Healthcare professionals, particularly those working in primary healthcare, play a critical role in influencing vaccination behaviors, as they serve as trusted sources of information and role models for the general population. However, evidence suggests that a proportion of healthcare professionals themselves exhibit vaccine hesitancy. For instance, approximately 15% of healthcare workers have been reported to be hesitant toward COVID-19 vaccination [12], while more than 30% demonstrate hesitancy toward influenza vaccination [13]. Given their influence on public attitudes, hesitancy among healthcare professionals may contribute to reduced vaccination uptake in the general population and among high-risk groups [14,15].

Vaccination behavior among healthcare professionals is shaped by multiple factors. Motivators of vaccine acceptance include perceived effectiveness in reducing disease burden and protecting oneself and others, including vulnerable individuals [16]. Conversely, hesitancy is often driven by concerns about vaccine safety and effectiveness, as well as perceptions that influenza is not a serious illness [17]. These attitudes are further influenced by contextual factors such as workplace environment, professional role, and cultural setting [18].

Understanding the determinants of vaccination attitudes and behaviors among healthcare professionals is essential for identifying gaps and developing targeted interventions. Therefore, the present study aims to investigate the beliefs, attitudes, and behaviors of primary healthcare professionals regarding influenza vaccination in Greece, as well as to identify factors associated with vaccination uptake.

## 2. Materials and Methods

### 2.1. Study Design and Setting

A cross-sectional study was conducted to assess beliefs, attitudes, and behaviors toward influenza vaccination, as well as vaccination advocacy among healthcare professionals in Greece. Data was collected using a self-administered, structured questionnaire. The study population comprised physicians and pharmacists providing care in primary healthcare settings, including private medical practices, health centers, and community pharmacies, across multiple regional health authorities.

### 2.2. Participants and Data Collection

A total of 304 healthcare professionals working in primary healthcare settings participated in the study. Eligible participants included physicians and community pharmacists actively practicing in primary healthcare settings across the seven Regional Health Authorities of Greece. More specifically, physicians were required to practice in primary healthcare settings, including general practitioners, internists, and other physicians providing primary care services, while pharmacists were required to work in community pharmacies where vaccination services are provided.

The questionnaire was disseminated electronically through the Hellenic Medical Association and the Panhellenic Pharmaceutical Association, which distributed the survey invitation to their registered members meeting the study criteria. The survey was sent to approximately 3300 physicians and 6000 community pharmacists. Data collection was conducted through the Microsoft Forms platform using an anonymous self-administered questionnaire. Participation was voluntary, and electronic informed consent was obtained prior to participation.

To ensure eligibility, the questionnaire included screening questions regarding educational level, professional status, medical specialty, workplace setting, and regional health authority. Participants not meeting the inclusion criteria, including healthcare professionals outside primary healthcare settings, non-physician/non-pharmacist respondents, or hospital-based professionals, were excluded from participation. Incomplete questionnaires were also excluded from the analysis.

The study aimed to achieve broad geographical representation across all seven Regional Health Authorities of Greece. The regional distribution of respondents was considered proportionate to the population distribution of healthcare professionals across the country. The professional composition of the sample was also considered relevant to vaccination practices in Greece, where both physicians and community pharmacists are authorized to administer influenza vaccines.

The questionnaire required approximately 10 min to complete. No personal identifiers, including names or telephone numbers, were collected in order to ensure anonymity and confidentiality. Participants were informed that all provided information would remain strictly confidential and would be used exclusively for scientific purposes in accordance with research ethics principles. Motivation toward influenza vaccination and vaccination advocacy were assessed using the validated MoVac-flu and MovAd scales, respectively. The questionnaire is provided in Supplementary Material S1.

### 2.3. Measurement Instruments

Motivation toward influenza vaccination (MoVac-flu)

Motivation toward influenza vaccination was assessed using the MoVac-flu scale, a validated instrument measuring motivational dimensions related to vaccination behavior. The scale consists of 9 items rated on a 7-point Likert scale (1 = strongly disagree to 7 = strongly agree), with higher scores indicating greater motivation.

The original validation study supported a one-factor structure [19], whereas the Greek validation study identified two factors: Vax Self-Care (items q1–q6), reflecting the perceived importance of vaccination for both individual health and the protection of others, and Vax Efficiency (items q7–q9), reflecting perceived knowledge and autonomy in vaccination-related decision-making [20].

Vaccination advocacy (MovAd)

Vaccination advocacy was assessed using the MovAd scale, a validated instrument evaluating healthcare professionals’ attitudes toward promoting vaccination. The scale includes 11 items rated on a 7-point Likert scale (1 = strongly disagree to 7 = strongly agree), with higher scores indicating stronger advocacy.

The original validation study supported a one-factor structure [19], while the Greek validation study identified two factors: Vax Cognitive Skills (items q1–q6), capturing the perceived importance and impact of discussing vaccination, and Vax Communication Skills (items q7–q11), reflecting confidence, knowledge, and autonomy in vaccination-related communication [20].

A pilot study was not conducted, as previously validated and culturally adapted instruments were used [20].

### 2.4. Confirmatory and Exploratory Factor Analyses, Internal Consistency and Validity Evaluation

Confirmatory factor analysis (CFA) was performed to evaluate whether the previously reported one- and two-factor structures of the MoVac-flu and MovAd scales could be replicated in the present sample. Given the ordinal nature of the items (7-point Likert scale), the robust weighted least squares estimator (WLSMV) was used. Model evaluation was based on multiple fit indices, including the Comparative Fit Index (CFI), Tucker–Lewis Index (TLI), Root Mean Square Error of Approximation (RMSEA), and Standardized Root Mean Square Residual (SRMR). Factor loadings were examined to ensure that items adequately represented the latent constructs. CFA was conducted in R statistical software (v4.3.2) [21] with RStudio integrated development environment (2023) [22] using the *lavaan* package.

Exploratory factor analysis (EFA) was conducted when the predefined factor structures did not adequately fit the study data [23]. Sampling adequacy was assessed using the Kaiser–Meyer–Olkin (KMO) measure with a cut-off of 0.50. Bartlett’s test of sphericity was also performed. Factors were extracted using the principal component method based on the correlation matrix and rotated using promax rotation with Kaiser normalization. The number of factors retained was determined based on eigenvalues (>1.0), the scree plot, and interpretability criteria. Factor loadings >0.40 were considered meaningful. Factor scores were calculated as the mean of the items comprising each factor. EFA was performed using SPSS version 23.0 [24].

Reliability was evaluated through internal consistency, which was assessed using Cronbach’s alpha coefficient [25]. Construct validity was evaluated through convergent and discriminant validity. Convergent validity was assessed using corrected item–total correlations, while discriminant validity was examined through inter-factor correlations in relation to Cronbach’s alpha coefficients. Spearman’s correlation coefficient was used to quantify the strength of associations.

### 2.5. Statistical Analysis

Descriptive statistics were used to summarize demographic, professional, and vaccination-related characteristics. Categorical variables are presented as frequencies and percentages, whereas continuous variables are presented as means with standard deviations (SD) or medians with interquartile ranges (IQR), as appropriate according to their distribution.

Comparative analyses were performed to examine differences according to influenza vaccination uptake during the 2023–2024 season, annual influenza vaccination behavior, and age group. Group differences in continuous variables were assessed using the independent-samples t test or non-parametric tests (Mann–Whitney U test, Kruskal–Wallis test), as appropriate. Associations between categorical variables were examined using the chi-square test or Fisher’s exact test.

To identify predictors of influenza vaccination behavior, univariable logistic regression analyses were first performed. Variables associated with the outcomes at a significance level of α = 10% were subsequently entered into multivariable logistic regression models to account for potential confounding. Two separate multivariable models were constructed, with influenza vaccination uptake during the 2023–2024 season and annual influenza vaccination as the dependent variables. Multicollinearity was assessed prior to model fitting using variance inflation factor (VIF), and no evidence of significant collinearity among independent variables was identified (VIF < 5 for all variables). Results are presented as odds ratios (ORs) and adjusted odds ratios (aORs) with 95% confidence intervals (CIs). Statistical significance was set at *p* < 0.05. All statistical analyses were performed using Stata 14.2 [26].

An a priori sample size calculation was not performed because the study was exploratory and based on voluntary nationwide participation through professional associations. Nevertheless, the sample achieved was adequate for the performed factor analyses, as well as for the multivariable logistic regression analyses.

## 3. Results

### 3.1. Demographic, Professional and Vaccination-Related Characteristics of the Study Population

Demographic, professional and vaccination-related characteristics of the study population are presented in Table 1. The study sample consisted of 304 healthcare professionals working in primary healthcare settings, of whom 52.6% were female and 47.4% male. Most participants were aged 41–60 years (57.6%), while 24% were aged 20–40 years and 18.4% were older than 60 years. Approximately two-thirds of participants reported having children (66.8%), and nearly three-quarters had parents aged over 65 years (74.0%). Almost 30% of the participants reported living with vulnerable individuals, and 62.2% taking care of them regularly.

Regarding professional characteristics, physicians accounted for 61.2% of the sample and pharmacists for 38.8%. Among physicians, internal medicine specialists represented the largest subgroup (50.6%), followed by general practitioners (43.0%). Participants worked across a variety of primary healthcare settings, mainly including private medical practices (40.5%), community pharmacies (34.9%), and health centers (9.5%). Although participants were distributed across all seven regional health authorities, most of them reported working on the 1st (Attica, 39.5%) and the 4th (Macedonia and Thrace, 19.4%) ones. Nearly 40% reported more than 20 years of professional experience.

Influenza vaccination uptake during the 2023–2024 season was reported by 77.6% of participants, while 75.3% reported receiving the influenza vaccine annually. Almost all participants reported not belonging to a high-risk group requiring vaccination based on the National Vaccination Program recommendations (98.4%).

### 3.2. Factor Analyses Results and Internal Consistency and Validity of the Derived Factors

Due to differences between the present sample and those of the original [19] and Greek [20] validation studies, the factor structure, reliability and validity were re-evaluated.

In the original validation study, both the MoVac-flu and MovAd scales were found to have a one-factor structure [19]. However, CFA of this unidimensional model yielded very poor fit indices in the present sample, indicating that a single-factor solution was not appropriate for either the MoVac-flu (robust CFI = 0.689, robust TLI = 0.585, robust RMSEA = 0.320, SRMR = 0.098) or the MovAd (robust CFI = 0.457, robust TLI = 0.302, robust RMSEA = 0.389, SRMR = 0.168) scale in our data.

In the Greek validation study, both the MoVac-flu and MovAd scales were found to have a two-factor structure [20]. The CFA provided support only for the proposed two-factor model for the MoVac-flu scale. The model demonstrated acceptable fit according to several indices (robust CFI = 0.950, robust TLI = 0.930, SRMR = 0.040), although the robust RMSEA was relatively elevated (0.140). All items loaded significantly on their hypothesized latent factors. Items q1-q6 exhibited moderate to strong standardized loadings on factor 1 (Vax Self-Care, range: 0.61–0.95), while items q7 and q8 showed strong loadings on factor 2 (Vax Efficiency, both > 0.95). Item q9 demonstrated a comparatively lower but statistically significant standardized loading (0.37). The Greek validation study labelled the second factor as “Vax Efficiency”; however, based on the content of the items and their conceptual meaning, we consider “Vax Awareness” to be a more accurate label. Therefore, we refer to this factor as “Vax Awareness” throughout the present study.

The MoVac-flu scale demonstrated high internal consistency, with a Cronbach’s alpha coefficient of 0.879 (Table 2). Internal consistency was also high for the Vax Self-Care factor (α = 0.892). The Vax Awareness factor showed lower internal consistency (α = 0.637) compared with the Vax Self-Care subscale. This may be related to the comparatively weaker loading of item q9. By removing item q9 from this factor, the Cronbach’s alpha value would increase from 0.637 to 0.922. However, item q9 was retained because it represents an important component of the original theoretical framework of the MoVac-flu scale, demonstrated a statistically significant, although comparatively lower, standardized loading in this factor, and its removal would have reduced the factor to only two items (q7 and q8), limiting its interpretability and stability. Regarding validity, the corrected item-total correlation was >0.30 for all items of the two factors, with one item (q9) showing a marginally acceptable value (0.22), indicating satisfactory convergent validity for both factors. The correlation between the two latent factors was moderate (r = 0.52), indicating related but distinguishable dimensions and supporting discriminant validity.

On the other hand, the two-factor model for the MovAd scale proposed by the Greek validation study was not supported by the CFA. Specifically, applying this model to the current sample resulted in poor fit indices (robust CFI = 0.639, robust TLI = 0.538, robust RMSEA = 0.280, SRMR = 0.151), indicating that it did not adequately capture the underlying structure of the items. Therefore, an EFA was conducted to examine the factor structure of the MovAd scale in the present sample. Sampling adequacy was confirmed by the KMO measure (0.836, exceeding the 0.50 threshold) and Bartlett’s test of sphericity (*p* < 0.001), indicating that the data were suitable for factor analysis. All items demonstrated high communalities (>0.70), and therefore no items were excluded. Evaluation of eigenvalues, the scree plot, and additional retention criteria supported the extraction of four factors, interpreted as “Vax Communication”, “Vax Influence”, “Vax Confidence”, and “Vax Choice”. All items loaded strongly on their respective factors, with factor loadings much above the recommended value of 0.40. Specifically, items q1–q3 exhibited strong factor loadings on Vax Communication factor (range: 0.93–0.95), items q4–q6 on Vax Influence factor (range: 0.77–0.95), items q7–q9 on Vax Confidence factor (range: 0.86–0.97), and items q10 and q11 on Vax Choice factor (both > 0.90). These four factors accounted for 85% of the total variance.

In contrast to the two-factor model for the MovAd scale proposed by the Greek validation study, the four-factor model suggested by EFA demonstrated excellent fit (robust CFI = 0.960, robust TLI = 0.942, RMSEA = 0.041, SRMR = 0.035), suggesting a more accurate representation of the latent constructs in this sample and preserving the multidimensional structure of the measurement. Despite some factors being defined by only 2–3 items, the factor loadings were substantial and statistically significant (0.77–0.97), supporting the validity of this structure. Therefore, the four-factor model was retained over the two-factor alternative due to superior model fit. This model provides a more reliable and meaningful representation of the construct for this population.

The MovAd scale demonstrated high internal consistency, with a Cronbach’s alpha coefficient of 0.873. Subscale internal consistency was very good for all subscales ranging between 0.817 and 0.934 (Table 3). As for validity, the corrected item-total correlation was >0.30 for all items of the four factors indicating a satisfactory convergent validity for all of them. The factor correlations ranged from 0.24 to 0.56, supporting discriminant validity.

### 3.3. Descriptive Statistics of Motivation Toward Influenza Vaccination and Vaccination Advocacy

Overall motivation toward influenza vaccination was high among participants. The mean total MoVac-flu score was 6.1 (SD = 0.83), with a median of 6 and a narrow IQR, indicating generally strong agreement with positive vaccination statements (Table 2). Participants reported particularly high scores on items related to the perceived value and impact of influenza vaccination (Vax Self Care: mean = 6.0; SD = 0.98), including its contribution to personal health, well-being, and protection of others. Scores related to perceived knowledge about vaccination protection were also high (Vax Awareness: mean = 6.3; SD = 0.79), and with slightly lower variability (Table 2).

The mean total MovAd score was 5.8 (SD = 0.76), with a median of 6 and a narrow IQR, reflecting positive attitudes toward vaccination advocacy and communication (Table 3). Participants reported high confidence in their ability to discuss vaccination and answer questions (Vax Confidence: mean = 6.0, SD = 0.89) and in their autonomy on discussing vaccination (Vax Choice: mean = 5.5, SD = 1.28), as well as a strong perception that such discussions can positively influence others’ beliefs and decisions (Vax Influence: mean = 5.5; SD = 1.03) and that vaccination is an important topic to discuss openly with others (Vax Communication: mean = 6.1, SD = 1.03) (Table 3).

### 3.4. Age and Influenza Vaccination Perspectives

Age-related differences were observed in vaccination attitudes. Detailed subscale distributions are presented in Supplementary Table S1. Regarding motivation toward influenza vaccination, median Vax Self-Care scores were significantly higher in older age groups (*p* = 0.028), whereas Vax Awareness scores did not differ significantly across groups (*p* = 0.555); however, they were also higher among participants aged > 40 years old. In terms of vaccination advocacy, significant age-related increases were observed for Vax Influence and Vax Confidence subscales (both *p* < 0.001), as well as for Vax Choice (*p* = 0.024). Differences in Vax Communication scores were not statistically significant (*p* = 0.055); however higher median scores were observed among older participants (Supplementary Table S1). Overall, older age was associated with more favorable motivation and advocacy-related subscale scores.

### 3.5. Differences According to Influenza Vaccination Uptake (2023–2024) and Annual Influenza Vaccination

Healthcare professionals vaccinated against influenza during the 2023–2024 season demonstrated higher motivation and advocacy scores compared with non-vaccinated participants (Table 4). Specifically, mean scores for both subscales of the MoVac-flu were significantly higher among vaccinated individuals (*p* < 0.001). Similarly, vaccinated participants exhibited significantly higher scores in all MovAd subscales (*p* < 0.05), except for Vax Choice (*p* = 0.699). All the above indicate that non-vaccinated participants exhibited consistently lower motivation toward influenza vaccination and weaker vaccination advocacy skills compared with vaccinated participants. Significant associations were also observed for several demographic variables, including age group, parental status, cohabitation with vulnerable individuals, and years of work experience. Regarding age, older age was significantly associated with vaccination against influenza during 2023–2024 (*p* < 0.001). A non-significant association was observed regarding sex (*p* = 0.058). (Table 4).

Consistent findings were observed when annual influenza vaccination behavior was examined. Participants reporting annual vaccination had higher motivation toward influenza vaccination and stronger advocacy-related skills than those who did not vaccinate annually (Table 5). However, participants reporting annual vaccination had comparable scores in the Vax Influence and Vax Choice subscales with those who did not (*p* = 0.111 and *p* = 0.627, respectively). Male sex, older age, having children, and longer professional experience were significantly associated with annual vaccination behavior. A non-significant association was observed regarding cohabitation with vulnerable individuals (*p* = 0.056). (Table 5).

### 3.6. Predictors of Influenza Vaccination Uptake (2023–2024) and Annual Influenza Vaccination Behavior

In univariable logistic regression analyses, higher scores on the subscales of MoVac-flu, as well as higher scores on the subscales of MovAd, were significantly associated with influenza vaccination uptake, except for Vax Choice (Table 6). Several demographic variables, including sex, age, having children, living with vulnerable individuals, workplace and years of work experience, were also associated with vaccination at a significance level of α = 10% and therefore they were subsequently included in the multivariable model to control for potential confounders. In this model, Vax Self-Care subscale emerged as a strong independent predictor of vaccination uptake, with higher scores (i.e., higher motivation) being significantly associated with higher odds of vaccination (aOR = 3.66, 95% CI: 2.33–5.77, *p* < 0.001). Additionally, living with individuals belonging to vulnerable groups remained independently associated with higher odds of vaccination (aOR = 2.95, 95% CI: 1.18–7.38, *p* = 0.020). Male sex and older age (>60 years) showed higher odds of vaccination (aOR = 2.08, 95% CI: 0.99–4.37, *p* = 0.054, and aOR = 6.70, 95% CI: 0.98–45.9, *p* = 0.053, respectively), but these associations were not statistically significant (Table 6).

Regarding annual vaccination, in univariable logistic regression analyses, apart from Vax Influence and Vax Choice, higher scores on all other subscales of MoVac-flu and MovAd were significantly associated with annual vaccination (Table 7). The same was observed for several demographic variables (sex, age, having children, living with vulnerable individuals, workplace and years of work experience). In the multivariable model, higher scores on Vax Self-Care (aOR = 3.22, 95% CI: 2.08–4.96, *p* < 0.001) and Vax Communication (aOR = 1.64, 95% CI: 1.14–2.34, *p* = 0.007) subscales, reflecting higher motivation and vaccination advocacy, respectively, as well as male sex (aOR = 2.35, 95% CI: 1.14–4.83, *p* = 0.020) remained independently associated with higher odds of annual influenza vaccination. Furthermore, older age (>60 years) showed higher odds of vaccination (aOR = 5.37, 95% CI: 0.88–32.7, *p*-value = 0.068), but this association was not statistically significant (Table 7).

## 4. Discussion

This study provides a comprehensive assessment of attitudes, beliefs, and behaviors of primary healthcare professionals toward influenza vaccination in Greece. Influenza vaccination uptake during the 2023–2024 season was relatively high, with approximately three-quarters of participants reporting vaccination. This proportion appears higher than that reported in several European and regional studies [16,27,28], potentially reflecting the focus on primary healthcare professionals, who are more actively engaged in preventive care and vaccination practices. Consistent with existing literature, vaccinated participants demonstrated significantly higher levels of motivation toward influenza vaccination and stronger vaccination advocacy compared with non-vaccinated participants [29,30,31]. Although previous validation studies supported one- or two-factor structures for the MoVac-flu and MovAd scales assessing motivation toward influenza vaccination and vaccination advocacy, respectively, the confirmatory factor analysis in our sample did not support these previously proposed models, particularly for the MovAd scale. Consequently, exploratory factor analysis was performed, revealing a four-factor structure. These findings suggest that the factorial structure of vaccination advocacy may vary across populations and healthcare settings, highlighting the importance of contextual validation when these instruments are applied in different groups. In addition, demographic characteristics, including sex and cohabitation with vulnerable individuals, seem to play a role in vaccination uptake. These are consistent with previous studies identifying similar determinants of vaccination behavior among healthcare professionals [31,32,33].

Differences were observed between seasonal (2023–2024) and annual vaccination behavior. While cohabitation with vulnerable individuals was associated with seasonal vaccination uptake, this association was not retained for annual vaccination. This pattern may reflect differences between contextual and more stable determinants of behavior, suggesting that certain influences are more prominent in specific contexts or timeframes. Context-dependent factors, such as perceived risk to close contacts, may play a greater role in shaping short-term vaccination decisions, particularly when the perceived threat is immediate or personally salient. In contrast, long-term behaviors appear to be driven more strongly by stable motivational and attitudinal constructs, which are less sensitive to changing circumstances. These findings highlight the dynamic nature of vaccination behavior and suggest that different mechanisms may underlie decisions depending on whether the behavior is examined over a shorter or longer time horizon [29,30,34,35].

Multivariable analysis identified vaccine self-care as a consistent and strong independent predictor of both seasonal and annual vaccination. This finding highlights the importance of intrinsic motivational factors, reflecting perceived personal and public health benefits of vaccination, and suggesting a more stable influence on behavior over time. In contrast, cohabitation with vulnerable individuals was associated only with seasonal vaccination uptake, indicating that external or contextual influences may have a more limited or transient impact. While concern for others may encourage vaccination in specific contexts, it may not sustain long-term adherence in the absence of stronger intrinsic motivation. Similar patterns have been reported in other vaccination contexts, where intrinsic motivation appears to be a more stable determinant than context-specific risk perceptions [36,37,38,39,40].

Beyond influenza, previous evidence suggests that cohabitation with high-risk individuals may also influence COVID-19 vaccination behavior. The 2023–2024 influenza season may therefore have been affected by broader pandemic-related changes in vaccination attitudes and risk perceptions, particularly among primary healthcare professionals. Although our study did not directly measure COVID-19-related perceptions or experiences, it is plausible that pandemic-associated changes in health behavior may have influenced influenza vaccination acceptance. This should be considered when interpreting the findings for the 2023–2024 season [36,37].

For annual vaccination, both vaccine-related communication skills and male sex were found to be independently associated with higher vaccination uptake. The association with communication-related competencies suggests that healthcare professionals who feel more confident and effective when discussing vaccination with primary health care users may also be more likely to adopt preventive health behaviors themselves. This pattern points to a link between professional practice and personal behavior, where confidence in promoting vaccination may reinforce adherence to it in one’s own life. It may also reflect greater awareness of vaccine benefits and a stronger alignment between knowledge and action.

In addition, the observed association with male sex is consistent with findings reported in previous studies examining influenza vaccination uptake and vaccine attitudes among healthcare professionals and adult populations [41,42]. However, the evidence across the literature remains inconsistent, with some studies reporting no significant differences or even contrasting results. Therefore, this finding should be interpreted with caution, and further research is needed to better understand the factors underlying these variations [31,32,33].

Overall, these findings support the distinction between stable and contextual determinants of vaccination behavior. Intrinsic motivational factors, such as vaccine self-care, appear to represent more robust and consistent predictors, as they appear to be rooted in enduring beliefs, values, and personal attitudes toward health and prevention. In contrast, contextual influences, including cohabitation with vulnerable individuals, may exert a more variable effect depending on specific circumstances and timeframes. This distinction has important implications for intervention design, suggesting that strategies aiming to improve vaccination uptake among healthcare professionals should address both intrinsic motivational factors and contextual influences. By fostering a deeper sense of personal responsibility and reinforcing the perceived value of vaccination, such approaches may lead to longer-lasting behavioral change. At the same time, addressing contextual barriers remains relevant, as these factors can either facilitate or hinder the translation of motivation into action in everyday practice.

Several limitations of this study should be considered. First, the study sample was predominantly drawn from large urban centers, which may limit the generalizability of the findings to rural or less-resourced settings where access to healthcare, vaccination programs, and health communication may differ significantly. Second, although both physicians and pharmacists were included in the study, the sample was largely composed of physicians. Other primary healthcare professionals, such as community health professionals and nurses, whose vaccination behaviors and influencing factors might differ, were not included in the study sample. Therefore, the observed associations between motivational factors and vaccination uptake may primarily reflect the perspectives and behaviors of primary healthcare professionals practicing predominantly in urban settings and may not be fully generalizable to rural healthcare settings or to other healthcare professional groups not included in the present study. Third, vaccination status and related variables were self-reported, which may introduce reporting bias, including over- or underestimation of uptake, and could affect the reliability of the observed associations. In addition, the study did not include variables directly assessing the potential influence of the COVID-19 pandemic on influenza vaccination attitudes and behaviors. Therefore, residual confounding related to pandemic-associated changes in vaccination perceptions and practices cannot be excluded. Finally, the cross-sectional design of the study prevents causal inferences, meaning that while associations can be identified, the directionality of the relationships cannot be firmly established.

Future research should aim to include more geographically and professionally diverse samples to improve representativeness and enable more detailed subgroup analyses across different healthcare settings and provider types. Expanding the sample in this way would help clarify whether observed patterns hold across rural, urban, and resource-limited contexts, as well as among various professional groups beyond physicians. In addition, longitudinal study designs would provide valuable insight into the stability of vaccination-related behaviors over time and allow for a clearer understanding of the relative influence of intrinsic versus contextual determinants on both short-term and long-term vaccination uptake. Such approaches could help identify which factors are most predictive of sustained preventive behaviors and inform the development of more targeted and effective intervention strategies.

A strength of this study is that it focused both on seasonal vaccination of 2023–2024 and annual vaccination. This is an important advantage because changes in beliefs, attitudes and behaviors are influenced constantly. It is impossible to reach safe conclusions just from the vaccination percentages of one season only. Moreover, when annual vaccination is included as a topic of study, it is possible to observe results on how beliefs and attitudes change overtime, how behaviors are ultimately shaped and what exactly are the factors which shape those behaviors. Additionally, the study involved a considerable number of participants, which helps support the results.

## 5. Conclusions

Influenza vaccination behavior among primary healthcare professionals in Greece is shaped by multiple interacting factors. Vaccination uptake was high for both the 2023–2024 season and annual vaccination, reflecting generally positive attitudes toward preventive care within this population. “Vaccine self-care” emerged as a consistent and strong predictor of vaccination, underscoring the role of intrinsic motivation in guiding health behaviors. In contrast, cohabitation with vulnerable individuals was associated only with seasonal uptake, suggesting that certain influences may be more context-dependent and exert a transient effect. These findings highlight the importance of intrinsic motivational determinants over situational influences in shaping vaccination behavior. Interventions that focus on enhancing motivation, improving knowledge, and strengthening communication skills among healthcare professionals may therefore lead to more sustained improvements not only in personal vaccination uptake but also in vaccine advocacy for primary health care users and the broader public with whom primary healthcare professionals interact. Expanding such initiatives more broadly across different regions and professional groups is essential to ensure that primary prevention strategies achieve their full potential and contribute to overall public health goals.

## Figures and Tables

**Table 1:** Demographic, professional and vaccination-related characteristics of the study population (N = 304).

Characteristic	Number	Percentage (%)
Sex		
Male	144	47.4
Female	160	52.6
Other	0	0.0
No answer	0	0.0
Age group (in years)		
20–40	73	24.0
41–60	175	57.6
>60	56	18.4
No answer	0	0.0
Having children		
Yes	203	66.8
No	99	32.6
No answer	2	0.6
Having parents aged >65 years		
Yes	225	74.0
No	79	26.0
No answer	0	0.0
Living with people of vulnerable groups		
Yes	87	28.6
No	216	71.1
No answer	1	0.3
Taking care of people of vulnerable groups regularly		
Yes	189	62.2
No	113	37.2
No answer	2	0.6
Profession		
Physician	186	61.2
Pharmacist	118	38.8
Specialty (for 186 physicians)		
General practitioner	80	43.0
Internal Medicine Physician (Internist)	94	50.6
Cardiologist	1	0.5
Pulmonologist	1	0.5
Paediatrician	3	1.6
Other	7	3.8
Main place of work		
Private medical practice	123	40.5
Health Center	29	9.5
Municipal Clinic	4	1.3
Community Pharmacy	106	34.9
Diagnostic Center	3	1.0
Other	39	12.8
Regional Health Authority		
1st (Attica)	120	39.5
2nd (Piraeus and Aegean Islands)	24	7.9
3rd (Western Macedonia)	15	4.9
4th (Macedonia and Thrace)	59	19.4
5th (Thessaly and Sterea)	23	7.6
6th (Peloponnese, Ionian Islands, Epirus and Western Greece)	45	14.8
7th (Crete)	18	5.9
Years of work experience		
<5	17	5.6
5–10	48	15.8
11–15	68	22.3
16–20	51	16.8
>20	120	39.5
Influenza vaccination uptake (2023–2024)		
Yes	236	77.6
No	67	22.1
No answer	1	0.3
Belonging to a high-risk group requiring vaccination based on the National Vaccination Program recommendations		
Yes	4	1.3
No	299	98.4
No answer	1	0.3
Annual influenza vaccination		
Yes	229	75.3
No	72	23.7
No answer	3	1.0

**Table 2:** List of items, dimensions and subscales of the MoVac-flu scale.

Scale/Subscale	Dimension	Item	Mean	Standard Deviation	Median	Interquartile Range	Cronbach’s Alpha
MoVac-flu			6.1	0.83	6	6–7	0.879
Vax Self Care			6.0	0.98	6.2	6–7	0.892
	Value						
	Q1	It is important that I get the flu jab	6.3	1.01	7	6–7	
	Q2	The contribution of the flu jab to my health and well-being is very important	6.1	1.19	6.5	5–7	
	Q3	The flu jab plays an important role in protecting my life and that of others	6.2	1.10	7	6–7	
	Impact						
	Q4	Vaccination is a very effective way to protect me against the flu	6.1	1.10	6	5–7	
	Q5	Vaccination greatly reduces my risk of catching the flu	5.5	1.56	6	5–7	
	Q6	Getting the flu jab has a positive influence on my health	5.8	1.26	6	5–7	
Vax Awareness (previously reported as “Vax Efficiency”)			6.3	0.79	6.3	6–7	0.637
	Knowledge						
	Q7	I know very well how vaccination protects me from the flu	6.4	0.91	7	6–7	
	Q8	I understand how the flu jab helps my body fight the flu virus	6.4	0.91	7	6–7	
	Autonomy						
	Q9	I can choose whether to get a flu jab or not	6.2	1.27	7	6–7	

**Table 3:** List of items, dimensions and subscales of the MovAd scale.

Scale/Subscale	Dimension	Item	Mean	Standard Deviation	Median	Interquartile Range	Cronbach’s Alpha
MovAd			5.8	0.76	6	5–6	0.873
Vax Communication			6.1	1.03	6.3	5–7	0.934
	Value						
	Q1	Vaccination is an important topic I want to discuss with others	5.9	1.18	6	5–7	
	Q2	It is important that I mention the topic of vaccination to others	6.2	1.05	7	6–7	
	Q3	It matters that I talk openly about vaccination with other people	6.1	1.07	6	5–7	
Vax Influence			5.5	1.03	5.7	5–6	0.865
	Impact						
	Q4	When I talk openly about vaccination, it has a positive impact on people’s beliefs	5.7	1.08	6	5–7	
	Q5	If I discuss vaccination, it will very much change others’ views on this topic	5.3	1.18	5	4–6	
	Q6	People’s opinions of vaccination can really be influenced by the conversations I have with them	5.4	1.21	5	5–6	
Vax Confidence			6.0	0.89	6	5–7	0.912
	Knowledge						
	Q7	I am confident I can answer questions that others could ask me about vaccination	6.1	0.96	6	6–7	
	Q8	I know exactly how to talk about vaccination with others	5.8	1.02	6	5–7	
	Q9	I feel able to discuss vaccination	6.1	0.91	6	6–7	
Vax Choice			5.5	1.28	5.5	5–7	0.817
	Autonomy						
	Q10	I decide whether to have conversations on vaccination with others	5.7	1.26	6	5–7	
	Q11	Discussing vaccination with others is entirely my choice	5.4	1.50	6	4–7	

**Table 4:** Differences in influenza vaccination uptake (2023–2024) across demographic, professional and vaccination-related characteristics.

	Influenza Vaccination Uptake (2023–2024)				
	No (n = 67)		Yes (n = 236)		*p*-Value
Characteristic	Number	Percentage (%)	Number	Percentage (%)	
MoVac-flu subscales					
Vax Self Care (mean, SD)	5.1	1.13	6.3	0.77	**<0.001**
Vax Awareness (mean, SD)	6.0	1.05	6.4	0.67	**<0.001**
MovAd subscales					
Vax Communication (mean, SD)	5.5	1.13	6.2	0.94	**<0.001**
Vax Influence (mean, SD)	5.2	1.03	5.5	1.02	**0.040**
Vax Confidence (mean, SD)	5.7	1.02	6.1	0.83	**0.016**
Vax Choice (mean, SD)	5.5	1.13	5.5	1.31	0.699
Sex					0.058
Male	25	37.3	119	50.4	
Female	42	62.7	117	49.6	
Age group (in years)					**<0.001**
20–40	27	40.3	46	19.5	
41–60	35	52.2	139	58.9	
>60	5	7.5	51	21.6	
Having children					**0.001**
Yes	34	50.7	169	72.2	
No	33	49.3	65	27.8	
Having parents aged >65 years					0.867
Yes	49	73.1	175	74.2	
No	18	26.9	61	25.8	
Living with people of vulnerable groups					**0.007**
Yes	10	15.1	76	32.2	
No	56	84.9	160	67.8	
Taking care of people of vulnerable groups regularly					0.133
Yes	36	54.5	152	64.7	
No	30	45.5	83	35.3	
Profession					0.804
Physician	42	62.7	144	61.0	
Pharmacist	25	37.3	92	39.0	
Specialty (for 186 physicians)					0.737
General practitioner	20	47.6	60	41.7	
Internal Medicine Physician (Internist)	19	45.3	75	52.1	
Other	3	7.1	9	6.2	
Main place of work					0.187
Private medical practice	22	32.9	101	42.8	
Health Center	8	11.9	21	8.9	
Community Pharmacy	22	32.8	83	35.2	
Other	15	22.4	31	13.1	
Regional Health Authority					0.090
1st (Attica)	29	43.3	90	38.1	
2nd (Piraeus and Aegean Islands)	8	11.9	16	6.8	
3rd (Western Macedonia)	0	0.0	15	6.4	
4th (Macedonia and Thrace)	15	22.4	44	18.7	
5th (Thessaly and Sterea)	7	10.4	16	6.7	
6th (Peloponnese, Ionian Islands, Epirus and Western Greece)	6	9.0	39	16.5	
7th (Crete)	2	3.0	16	6.8	
Years of work experience					**0.020**
<5	7	10.4	10	4.3	
5–10	17	25.4	31	13.1	
11–15	13	19.4	55	23.3	
16–20	11	16.4	39	16.5	
>20	19	28.4	101	42.8	
Belonging to a high-risk group requiring vaccination based on the National Vaccination Program recommendations					0.580
Yes	0	0.0	4	1.7	
No	66	100.0	232	98.3	

SD, standard deviation; Bold *p*-values indicate statistically significant differences at a significance level of α = 5%.

**Table 5:** Differences in annual (every-year) influenza vaccination across demographic, professional and vaccination-related characteristics.

	Annual Influenza Vaccination				
	No (n = 72)		Yes (n = 229)		*p*-Value
Characteristic	Number	Percentage (%)	Number	Percentage (%)	
MoVac-flu subscales					
Vax Self Care (mean, SD)	5.2	1.18	6.3	0.72	**<0.001**
Vax Awareness (mean, SD)	6.0	1.03	6.5	0.64	**<0.001**
MovAd subscales					
Vax Communication (mean, SD)	5.5	1.14	6.3	0.90	**<0.001**
Vax Influence (mean, SD)	5.3	1.04	5.5	1.02	0.111
Vax Confidence (mean, SD)	5.7	1.05	6.1	0.80	**<0.001**
Vax Choice (mean, SD)	5.6	1.11	5.5	1.31	0.627
Sex					**0.031**
Male	26	36.1	116	50.7	
Female	46	63.9	113	49.3	
Age group (in years)					**0.001**
20–40	28	38.9	45	19.6	
41–60	38	52.8	135	59.0	
>60	6	8.3	49	21.4	
Having children					**0.003**
Yes	38	52.8	164	71.9	
No	34	47.2	64	28.1	
Having parents aged >65 years					0.609
Yes	55	76.4	168	73.4	
No	17	23.6	61	26.6	
Living with people of vulnerable groups					0.056
Yes	14	19.7	72	31.4	
No	57	80.3	157	68.6	
Taking care of people of vulnerable groups regularly					0.207
Yes	40	56.3	148	64.6	
No	31	43.7	81	35.4	
Profession					0.779
Physician	43	59.7	141	61.6	
Pharmacist	29	40.3	88	38.4	
Specialty (for 184 physicians)					0.626
General practitioner	19	44.2	59	41.8	
Internal Medicine Physician (Internist)	20	46.5	74	52.5	
Other	4	9.3	8	5.7	
Main place of work					0.413
Private medical practice	25	34.7	98	42.8	
Health Center	6	8.4	21	9.2	
Community Pharmacy	26	36.1	79	34.5	
Other	15	20.8	31	13.5	
Regional Health Authority					0.255
1st (Attica)	31	43.1	88	38.4	
2nd (Piraeus and Aegean Islands)	7	9.7	16	7.0	
3rd (Western Macedonia)	1	1.4	14	6.1	
4th (Macedonia and Thrace)	17	23.6	42	18.3	
5th (Thessaly and Sterea)	7	9.7	16	7.0	
6th (Peloponnese, Ionian Islands, Epirus and Western Greece)	7	9.7	37	16.2	
7th (Crete)	2	2.8	16	7.0	
Years of work experience					**0.035**
<5	7	9.7	10	4.4	
5–10	18	25.0	30	13.1	
11–15	13	18.1	55	24.0	
16–20	12	16.6	38	16.6	
>20	22	30.6	96	41.9	
Belonging to a high-risk group requiring vaccination based on the National Vaccination Program recommendations					0.576
Yes	0	0.0	4	1.8	
No	72	100.0	225	98.2	

SD, standard deviation; Bold *p*-values indicate statistically significant differences at a significance level of α = 5%.

**Table 6:** Univariable and multivariate logistic regression models. Predictors of influenza vaccination uptake (2023–2024).

	Univariable Models ^1^			Multivariable Model ^2^		
Variable	Odds Ratio	95% Confidence Interval	*p*-Value	aOdds Ratio	95% Confidence Interval	*p*-Value
MoVac-flu subscales						
Vax Self Care	3.42	2.42–4.84	**<0.001**	3.66	2.33–5.77	**<0.001**
Vax Awareness	1.93	1.38–2.69	**<0.001**	0.89	0.51–1.53	0.667
MovAd subscales						
Vax Communication	1.87	1.43–2.45	**<0.001**	1.35	0.92–1.96	0.121
Vax Influence	1.32	1.01–1.71	**0.041**	0.78	0.51–1.20	0.265
Vax Confidence	1.55	1.15–2.10	**0.004**	1.02	0.60–1.73	0.942
Vax Choice	1.04	0.84–1.29	0.698	-	-	-
Sex						
Female	Ref	-	-	Ref	-	-
Male	1.71	0.98–2.98	**0.059**	2.08	0.99–4.37	0.054
Age group (in years)						
20–40	Ref	-	-	Ref	-	-
41–60	2.33	1.28–4.26	**0.006**	2.27	0.63–8.13	0.209
>60	5.99	2.13–16.8	**0.001**	6.70	0.98–45.9	0.053
Having children						
No	Ref	-	-	Ref	-	-
Yes	2.52	1.44–4.41	**0.001**	1.46	0.63–3.39	0.373
Having parents aged >65 years						
No	Ref	-	-	-	-	-
Yes	1.05	0.57–1.95	0.867	-	-	-
Living with people of vulnerable groups						
No	Ref	-	-	Ref	-	-
Yes	2.66	1.29–5.50	**0.008**	2.95	1.18–7.38	**0.020**
Taking care of people of vulnerable groups regularly						
No	Ref	-	-	-	-	-
Yes	1.53	0.88–2.65	0.134	-	-	-
Profession						
Physician	Ref	-	-	-	-	-
Pharmacist	1.07	0.61–1.88	0.804	-	-	-
Specialty (for physicians)						
General practitioner	Ref	-	-			
Internal Medicine Physician (Internist)	1.32	0.64–2.69	0.451	-	-	-
Other	1.00	0.25–4.06	0.999	-	-	-
Main place of work						
Private medical practice	Ref	-	-	Ref	-	-
Community Pharmacy	0.82	0.43–1.59	0.559	1.48	0.57–3.82	0.417
Health Center	0.57	0.22–1.46	0.242	0.57	0.16–2.05	0.391
Other	0.45	0.21–0.97	**0.042**	1.02	0.35–2.95	0.976
Regional Health Authority						
1st (Attica)	Ref	-	-	-	-	-
Non-1st	1.24	0.71–2.15	0.447	-	-	-
Years of work experience						
1–10	Ref	-	-	Ref	-	-
11–15	2.48	1.13–5.44	**0.024**	1.06	0.32–3.43	0.928
16–20	2.08	0.90–4.80	**0.088**	0.41	0.09–1.73	0.225
>20	3.11	1.54–6.28	**0.002**	0.85	0.21–3.49	0.818

^1^ Bold *p*-values indicate statistically significant associations at a significance level of α = 10%. ^2^ Bold *p*-values indicate statistically significant associations at a significance level of α = 5%.

**Table 7:** Univariable and multivariate logistic regression models. Predictors of annual (every-year) influenza vaccination.

	Univariable Models ^1^			Multivariable Model ^2^		
Variable	Odds Ratio	95% Confidence Interval	*p*-Value	aOdds Ratio	95% Confidence Interval	*p*-Value
MoVac-flu subscales						
Vax Self Care	3.49	2.47–4.94	**<0.001**	3.22	2.08–4.96	**<0.001**
Vax Awareness	2.10	1.50–2.95	**<0.001**	0.91	0.53–1.56	0.733
MovAd subscales						
Vax Communication	2.18	1.65–2.88	**<0.001**	1.64	1.14–2.34	**0.007**
Vax Influence	1.23	0.95–1.59	0.112	-	-	-
Vax Confidence	1.67	1.24–2.25	**0.001**	0.90	0.57–1.42	0.656
Vax Choice	0.95	0.77–1.17	0.626	-	-	-
Sex						
Female	Ref	-	-	Ref	-	-
Male	1.82	1.05–3.14	**0.032**	2.35	1.14–4.83	**0.020**
Age group (in years)						
20–40	Ref	-	-	Ref	-	-
41–60	2.21	1.22–4.00	**0.009**	2.16	0.62–7.47	0.225
>60	5.08	1.93–13.4	**0.001**	5.37	0.88–32.7	0.068
Having children						
No	Ref	-	-	Ref	-	-
Yes	1.88	1.24–2.85	**0.003**	1.15	0.51–2.59	0.729
Having parents aged >65 years						
No	Ref	-	-	-	-	-
Yes	0.85	0.46–1.58	0.609	-	-	-
Living with people of vulnerable groups						
No	Ref	-	-	Ref	-	-
Yes	2.75	0.98–3.57	**0.059**	1.45	0.65–3.25	0.361
Taking care of people of vulnerable groups regularly						
No	Ref	-	-	-	-	-
Yes	1.42	0.82–2.43	0.208	-	-	-
Profession						
Physician	Ref	-	-	-	-	-
Pharmacist	0.93	0.54–1.59	0.779	-	-	-
Specialty (for physicians)						
General practitioner	Ref	-	-			
Internal Medicine Physician (Internist)	1.19	0.58–2.44	0.631	-	-	-
Other	0.64	0.17–2.38	0.509	-	-	-
Main place of work						
Private medical practice	Ref	-	-	Ref	-	-
Community Pharmacy	0.78	0.42–1.45	0.424	1.52	0.62–3.72	0.361
Health Center	0.89	0.33–2.45	0.826	1.62	0.40–6.54	0.495
Other	0.53	0.25–1.12	**0.097**	1.50	0.53–4.30	0.447
Regional Health Authority						
1st (Attica)	Ref	-	-	-	-	-
Non-1st	1.21	0.71–2.07	0.484	-	-	-
Years of work experience						
1–10	Ref	-	-	Ref	-	-
11–15	2.64	1.21–5.79	**0.015**	1.42	0.45–4.47	0.552
16–20	1.98	0.87–4.49	0.102	0.53	0.13–2.18	0.380
>20	2.73	1.38–5.39	**0.004**	0.99	0.25–3.94	0.984

^1^ Bold *p*-values indicate statistically significant associations at a significance level of α = 10%. ^2^ Bold *p*-values indicate statistically significant associations at a significance level of α = 5%.

## Data Availability

The data that support the findings of this study are available from the corresponding author upon reasonable request. The data is not publicly available due to privacy and ethical restrictions.

## Supplementary Materials

The following supporting information can be downloaded at: https://www.mdpi.com/article/10.3390/vaccines14060500/s1, Supplementary Material S1. Questionnaire. Supplementary Table S1. MoVac-Flu and MovAd subscale scores by age group.
