# Supplementary material for: Attitudes, Motivation, and Predictors of Influenza Vaccination Uptake Among Primary Healthcare Professionals in Greece
Source: Vaccines (Basel). 2026 Jun 2;14(6):500. doi: 10.3390/vaccines14060500 (PMC13307779; doi:10.3390/vaccines14060500)
Supplement: Supplementary file 1 [file vaccines-14-00500-s001.zip › vaccines-4330609-supplementary.pdf]

Supplementary Material

Supplementary Material S1. Questionnaire

This questionnaire was used to assess demographic characteristics, vaccination behavior, and validated constructs of motivation (MoVac-Flu) and advocacy (MovAd).

Questionnaire

1. Research Title – Institution – Principal Investigator
You are invited to participate in a scientific study titled: "Beliefs, attitudes, and behaviors of Primary Health Care Professionals regarding the effectiveness and efficiency of influenza vaccination in the community," conducted in the context of a doctoral dissertation of the Department of Public and Community Health of the University of West Attica. The Principal Investigator is Dr. Areti Lagiou, Professor of Epidemiology, Disease Prevention, and Public Health; Member of the Administrative Council of the University of West Attica; and Director of the Laboratory of Hygiene and Epidemiology of the Department of Public and Community Health, School of Public Health, University of West Attica.

2. What is the purpose of the study?
The purpose of this scientific study is to investigate the beliefs and attitudes of Primary Health Care Professionals regarding influenza vaccination and its promotion to the general population. The study is addressed to all Primary Health Care Professionals across the country.

3. Why have you been invited to participate?
You have been invited to participate in this study because you are a Primary Health Care Professional. In order to participate, you must:
a) be over 18 years of age,
b) speak and understand the Greek language,
c) have the capacity to provide consent.

4. What does your participation in the study involve?
During your participation in the study, you will be asked to answer questions concerning your demographic, professional, and social characteristics, as well as questions related to your relationship with seasonal influenza vaccination.

5. Are you obliged to participate?
Your participation in the study is entirely voluntary. You may refuse to participate without providing any reason or justification. You may change your mind at any time and withdraw from the study without any reason or justification and without any consequences for you. In this case, you may request that your data be deleted or that part of the processing be stopped. You may choose not to answer at all or to skip any questions in the questionnaire that follows.

6. Are there any potential risks from your participation in the study?
Your participation in the study does not involve any particular risk or financial burden for you.

7. Is there any benefit from your participation in the study?
There are no specific benefits from your participation in this study. Potential benefits include providing you with the opportunity to express your views to an objective researcher, and this information may help other individuals with similar problems or situations in the future.

8. Is there any compensation for participation?
No, no compensation is provided for your participation in the study, nor will you incur any cost.

9. How will your data be protected?
The storage of data and access to it will be carried out exclusively by the researchers of the study. All material from this study will be maintained in electronic form, anonymously, for a period of 3 years, and after the end of this three-year period, the electronic files will be permanently deleted. The processing of your personal data is based on your consent. Throughout the duration of the study, as well as thereafter, your personal information will remain coded (i.e., your name will not be used) and protected on an electronic computer. That is, for the analysis of the results, your name will not be used, but rather a code number, through which it will not be possible at any time during the study to identify your identity or personal details. You have the right to request from the Principal Investigator access to, correction, or deletion of your personal data, or restriction of its processing. In addition, you may object to its processing or exercise your right to data portability. You may withdraw from the study at any time. Your consent is given for 3 years or until it is withdrawn. If you withdraw your consent, all data collected will be deleted. The right to withdraw your consent at any time does not affect the lawfulness of the processing of personal data carried out based on consent prior to its withdrawal.

10. How can you contact us?
For further information, clarifications, or complaints regarding the study, please contact us at: ikougioumtzoglou@uniwa.gr.

11. Submission of complaints
For any complaint regarding the conduct of the study, you may contact the Research Ethics and Deontology Committee of the University of West Attica (ethics@uniwa.gr). For any complaint regarding the management of your personal data, you may also contact the Data Protection Officer of the University of West Attica, Mr. Dimitrios Bletsas (d.mpletsas@uniwa.gr). If your issue is not resolved, you may contact the Hellenic Data Protection Authority by completing the relevant form available on its website (complaints@dpa.gr).

12. Declaration of consent
I declare that I have been fully informed about the terms of my participation in the study and the processing of my personal data. I provide my explicit consent for my participation in the study and for the processing of personal data.

- Yes
- No

13. Gender:

- Male
- Female
- Other
- Prefer not to answer

14. Which age group do you belong to?

- 20–40 years
- 41–60 years

- Over 60 years
- Prefer not to answer

15. Do you have children?
- Yes
- No
- Prefer not to answer

16. Do you have elderly parent(s) (>65 years old)?
- Yes
- No
- Prefer not to answer

17. Do you live with individuals who belong to vulnerable groups?
- Yes
- No
- Prefer not to answer

18. Do you provide periodic or regular home care to individuals who belong to vulnerable groups (parents, children)?
- Yes
- No
- Prefer not to answer

19. What is the highest level of education you have completed?
- Technological education
- Bachelor's degree
- Master's degree
- PhD

20. What is your professional role?
- Physician
- Pharmacist
- Other

21. If you are a Physician, please specify your specialty:
- General Practitioner
- Internist
- Cardiologist
- Pulmonologist
- Pediatrician
- Other

22. What is your primary workplace?
- Private Practice
- Health Center

- Municipal Clinic
- Community Pharmacy
- Diagnostic Center
- Other

23. In which Health Region do you operate?

- 1st Health Region (Attica)
- 2nd Health Region (Piraeus and Aegean)
- 3rd Health Region (Western Macedonia)
- 4th Health Region (Macedonia and Thrace)
- 5th Health Region (Thessaly & Central Greece)
- 6th Health Region (Peloponnese, Ionian Islands, Epirus, and Western Greece)
- 7th Health Region (Crete)

24. How many years of professional experience do you have in your field?

- < 5 years
- 5–10 years
- 11–15 years
- 16–20 years
- > 20 years

25. Have you been vaccinated with the influenza vaccine during the current 2023–2024 flu season?

- Yes
- No
- Prefer not to answer

26. Do you belong to a high-risk group that requires vaccination according to the recommendations of the National Immunization Program (NIP)?

- Yes
- No
- Prefer not to answer

27. Do you receive the influenza vaccine annually?

- Yes
- No
- Prefer not to answer

28. Are you aware of the new generation influenza vaccines (High-Dose)?

- Yes
- No
- Prefer not to answer

29. If you answered "Yes" to question 28, what is your opinion on the effectiveness of the new vaccines?

- They are more effective than the existing ones
- They are equally effective as the existing ones

- They are less effective than the existing ones
- I have no opinion
- Prefer not to answer

30. If you answered "Yes" to question 28, what is your opinion on the safety of the new vaccines?

- They are safer than the existing ones
- They are equally safe as the existing ones
- They are less safe than the existing ones
- I have no opinion
- Prefer not to answer

31. Through which channels do you get information about changes and the current adult vaccination program?

- Pharmaceutical Companies
- Scientific Societies
- Medical Associations
- Ministry of Health – Hellenic National Public Health Organization (EODY)
- Patients
- Other

32. For what percentage of your patients who belong to a high-risk group do you prescribe the influenza vaccine without being asked?

- < 30%
- 30–75%
- > 75%

33. For what percentage of your patients who belong to a high-risk group do you prescribe the influenza vaccine after they request it?

- < 30%
- 30–75%
- > 75%

34. Regarding the adult vaccination program: How well informed do you consider yourself to be?

- Very Little
- Little
- Fairly
- Well
- Very Well

35. Which other vaccines from the National Adult Immunization Program (NIP) do you recommend, apart from the influenza vaccine?

- Tetanus, Diphtheria, Pertussis
- Measles, Mumps, Rubella
- Chickenpox
- Shingles (Herpes Zoster)
- Human Papillomavirus (HPV)

- Hepatitis A & B
- Pneumococcal
- Meningococcal
- Haemophilus influenzae

36. Responses were recorded on a 7-point Likert scale (1 = strongly disagree to 7 = strongly agree).
It is important for me to get the influenza vaccine.
The contribution of the influenza vaccine to my health and well-being is very important.
Influenza vaccination plays an important role in protecting my life as well as the lives of others.
Vaccination is a very effective way to protect myself from influenza.
Vaccination significantly reduces the risk of contracting influenza.
Getting the influenza vaccine has a positive impact on my health.
I am very knowledgeable about how influenza vaccination protects me from the flu.
I understand how influenza vaccination helps my body fight the virus.
I can choose whether or not to get vaccinated.

37. Responses were recorded on a 7-point Likert scale (1 = strongly disagree to 7 = strongly agree).
Vaccination is an important topic that I want to discuss with others.
It is important for me to bring up the topic of vaccination with others.
It matters to me to openly discuss vaccination with others.
When I discuss vaccination openly, it has a positive impact on the beliefs of others.
If I discuss vaccination, it will significantly influence the beliefs of others on the topic.
The opinions of others about vaccination can be significantly affected by the discussions I have with them.
I feel confident that I can answer questions others may ask me about vaccination.
I know exactly how to discuss vaccination with others.
I feel capable of discussing vaccination.
I decide whether or not to have discussions about vaccination with others.
Discussing vaccination with others is entirely my decision.

Supplementary Table S1. MoVac-Flu and MovAd subscale scores by age group

	**Age group (in years)**			**p-value**
**Characteristic**	**20-40 (n=73, 24.0%)**	**41-60 (n=175, 57.6%)**	**>60 (n=56, 18.4%)**	
MoVac-flu subscales				
Vax Self Care (median, IQR)	6 (5-6.5)	6.2 (5.5-7)	6.4 (5.8-7)	**0.028**
Vax Awareness (median, IQR)	6.3 (6-7)	6.7 (6-7)	6.7 (6-7)	0.555
MovAd subscales				
Vax Communication (median, IQR)	6 (5-7)	6.3 (5.7-7)	6.7 (5.5-7)	0.055
Vax Influence (median, IQR)	5 (4.3-5.7)	5.7 (5-6.3)	6 (5.3-6.3)	**<0.001**
Vax Confidence (median, IQR)	5.7 (5-6.3)	6 (5.7-7)	6.7 (5.7-7)	**<0.001**
Vax Choice (median, IQR)	5 (4.5-6)	5.5 (5-7)	6 (5-7)	**0.024**

Data are presented as median (IQR).
Bold values indicate statistical significance at $p < 0.05$.
